# The Genetic Architecture of Type 1 Diabetes

**DOI:** 10.3390/genes8080209

**Published:** 2017-08-22

**Authors:** Samuel T. Jerram, Richard David Leslie

**Affiliations:** Bart’s and the London School of Medicine and Dentistry, QMUL, London E1 2AT, UK; s.jerram@qmul.ac.uk

**Keywords:** Type 1, diabetes, genetic, genes

## Abstract

Type 1 diabetes (T1D) is classically characterised by the clinical need for insulin, the presence of disease-associated serum autoantibodies, and an onset in childhood. The disease, as with other autoimmune diseases, is due to the interaction of genetic and non-genetic effects, which induce a destructive process damaging insulin-secreting cells. In this review, we focus on the nature of this interaction, and how our understanding of that gene–environment interaction has changed our understanding of the nature of the disease. We discuss the early onset of the disease, the development of distinct immunogenotypes, and the declining heritability with increasing age at diagnosis. Whilst Human Leukocyte Antigens (HLA) have a major role in causing T1D, we note that some of these HLA genes have a protective role, especially in children, whilst other non-HLA genes are also important. In adult-onset T1D, the disease is often not insulin-dependent at diagnosis, and has a dissimilar immunogenotype with reduced genetic predisposition. Finally, we discuss the putative nature of the non-genetic factors and how they might interact with genetic susceptibility, including preliminary studies of the epigenome associated with T1D.

## 1. Introduction

**Figure 1:**
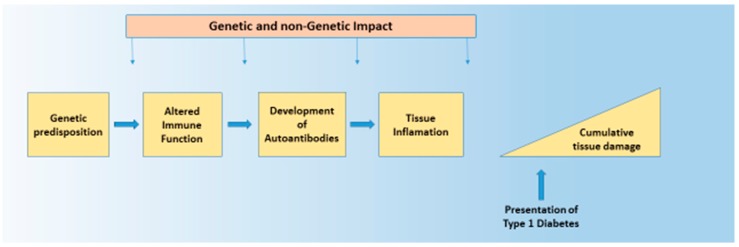
Evolution of pathogenicity over time. In patients with genetic disposition to disease, the clinical presentation of Type 1 diabetes (T1D) is predated by environmental exposure leading to changes in the immune system and development of autoantibodies. Note that the destruction of tissue—insulin producing cells in the case of T1D—predates the presentation of the illness, and continues after presentation also. Genetic impact can operate at different stages throughout the disease process.

**Figure 2:**
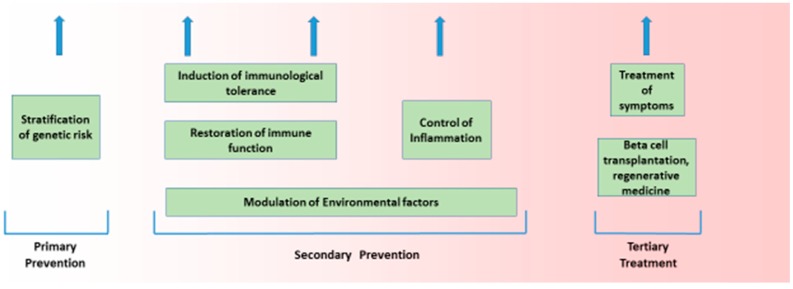
Potential therapeutic options in the prevention and treatment of T1D. Currently, the only options available are the treatment of symptoms and beta cell transplantation; however, in the future, genetic stratification may allow secondary preventative measures to be employed.

The aetiology of Type 1 diabetes (T1D), like most common chronic diseases, is complex and results from the interaction of genetic and environmental factors. That interplay takes place in a sequence that is true for all autoimmune diseases, and encompasses genetic susceptibility, tissue inflammation, and clinical disease [1,2]. This sequence is characterised by a diminished risk of progression at each transition, with more subjects having genetic risk (roughly 20%) than have inflammation (roughly 14%), and more having inflammation than have any autoimmune disease (roughly 7%). Genetic and non-genetic factors likely operate at all stages of this process. Such a structure is seen in T1D. The general features of this genetic and non-genetic interaction in T1D risk are discussed below, as that is the purpose of this broad review. The value of understanding the genetic origins of any disease is to delineate what is not genetic and therefore potentially reversible. Here, we discuss how recent developments in T1D can help us understand the architecture of the disease. (Figure 1 and Figure 2).

## 2. Structure of Disease Processes

Organ-specific autoimmune diseases involve a target-specific destruction leading to excess, or, more often, a deficiency of the cell-specific function of the target organ; in T1D, that process involves the destruction of the insulin-secreting cells of varying severity. The destructive process is thought to be immune-mediated with an autoimmune element. Potent evidence that the disease has an immunological basis comes from evidence that the principle genetic susceptibility resides in immune response genes and that other ‘autoimmune’ diseases share the same or similar genetic risk factors.

### 2.1. Heritability Is Variable

That T1D is genetically determined is illustrated by family, twin, and genetic studies. The frequency of T1D is higher in siblings of diabetic patients (e.g., in the UK 6% by age 30) than in the general population (in the U.K. 0.4% by age 30), while disease concordance in monozygotic (MZ) twins is about 40%, but is age at diagnosis dependent [1]. Even in children aged less than 5 years at diagnosis, the projected concordance rate in MZ twins is short of 100% at about 85% (though note the caveat below) [3]. The term heritability reflects gene expression or penetrance in a given environment. The best estimate of heritability can be obtained by determining the concordance rates of twins. Both identical and non-identical twins share the same environment in childhood, but only identical twins share, to a substantial degree, the same genes. In the classic twin method, the difference between the concordance rates for identical and non-identical twins is doubled to give an index of heritability. Higher concordance rates, for autoimmune diseases in general and T1D in particular, in identical compared with non-identical twins, are consistent with a genetic influence on these diseases. Estimates of heritability can be obtained from studies in Finland and the University of Southern California; in both studies, the estimates are substantially less than 100%, which means the disease is unlikely to be autosomal dominant. The problems with heritability estimates have been recently described [2,4]; they include problems with inference in that concordance between twins is impacted by shared environment as well as shared genes, including in utero environment, plus variability in genetic structure even between identical twins due to copy number variation and somatic mutations. However, the genetics of autoimmune diabetes shows substantial disease heterogeneity, and that variability is reflected in twin studies irrespective of these limitations of heritability estimates. A survival analysis estimated that non-diabetic identical twins of probands diagnosed with T1D under 25 years of age had, in one study, a 38% probability of developing diabetes compared with only 6% for twins of probands diagnosed later [1]. The three major studies of twins all found that the concordance rate for diabetes is highest in children and lowest in adults without any clear age of division [2]; moreover, all three studies found that the concordance rate, irrespective of age at diagnosis, was less than 100%, consistent with a non-genetically determined effect which becomes more prominent with increasing age at diagnosis. The remarkably low twin concordance rate for adult-onset T1D implies that the genetic impact in adult-onset T1D is limited, and certainly lower than that in childhood-onset disease. In contrast, the concordance rate in very young T1D twins is very high, with up to 85% of their co-twins developing diabetes [3]; importantly, in that study, the numbers of twins at follow-up beyond 60% concordance was very low with substantial confidence intervals, but the evidence that the concordance rate is high in such early onset T1DM twins is clear.

Age-related genetic factors influence the risk of T1D, but also the presence of diabetes-associated autoantibodies, the rate of progression to clinical diabetes, as well as the severity of reduced insulin secretory capacity. Not only is the age incidence of T1D lower in adults than in children, the range of incidence across European countries is also reduced. These age-related effects have been discussed by us in detail elsewhere [5]. Taken in conjunction with the variable disease heritability they imply differential risk factors, reflected in the broad range of age at diagnosis.

### 2.2. Age-Related Effects on Type 1 Diabetes Onset

Glutamic acid decarboxylase autoantibodies (GADA), when found in isolation, are more common than insulin autoantibodies (IAA) in Human Leukocyte Antigen (HLA)-DR3/3 children, but less common in HLA-DR4/8 children [6]. Diabetes-associated autoantibodies (DAA) can occur very early in life (for IAA the median age of appearance is 9 months), and the order of appearance is related to HLA-DR-DQ genotype. Two broad groups have been described: those with multiple autoantibodies, including IAA and insulinoma-associated-2 autoantibodies (IA2A), with a median age of diabetes onset aged 2 and a strong association with HLA-DR4-DQ8, and those with GADA as a single antibody, in whom the disease onset median is 4 years of age, but with a very slow progression to diabetes (if at all) and a genetic association with HLA-DR3-DQ2 [7]. Nevertheless, the lower genetic load in adult-onset T1D combined with subtle variation in the HLA class II gene associations and autoreactivity may account for or reflect the slower disease progression toward insulin deficiency in adults. Even in those cases with multiple autoantibodies in childhood, it can be demonstrated that their risk of progression to clinical diabetes is log-linear in nature, with an annual rate of conversion to clinical diabetes of 11% [8]. Moreover, the median age may be 6 years, but the progression is such that it can be estimated that finally 100% of cases with multiple autoantibodies in childhood will develop diabetes, and the age is about 60 years at which approximately 100% of cases develop the disease. By implication, 50% of these multiple autoantibody positive cases develop diabetes between the age of 6 and 60 years, and the near-certainty of that progression has led to the proposal that T1D be redefined to incorporate patients at genetic risk and with two or more DAA [8].

### 2.3. Age-Related Effect on Age at Diagnosis

A study of twins and siblings indicated that age at diagnosis was strongly genetically determined. In MZ twins concordant for T1D, the age at diagnosis was strongly correlated in 116 identical pairs (R = 0.94) and more closely than that in non-identical twins (R = 0.59) [9]. Since twins are diagnosed not only at the same time but also at the same age, we then performed a family study to estimate which was relevant. This study of siblings indicated that it was age at diagnosis and not time that dictated the strong correlation, and by implication disease risk was not due to increased risk at a given time but at a given age [9]. Thereafter, the rate of progression to clinical diabetes must be relatively constant, as reflected in the log-linear increase in disease risk shown in multiple antibody-positive children. That rate of decline could be genetically determined; however, the genes that could cause such an effect are unknown. It seems unlikely that we can account for the strong correlation with age over some 70 years simply by the effect of two clusters represented by associations with HLA haplotypes (either DR4-DQ8 or DR3-DQ2 or both).

In summary, given the evidence that the non-genetic events inducing T1D operate in early life and the age at diagnosis is, in part, genetically determined, it seems likely that there is a variable rate of progression of loss of insulin secretory capacity and that genetic factors impact that rate of decline. However, we have no idea precisely what dictates that variable rate of decline. Nevertheless, the variable age at diagnosis and the variable severity of loss of insulin secretory capacity, the younger the worse, points towards a spectral effect of declining disease aggression with increasing age, which is to be discussed below.

### 2.4. Evolution of Genetic Risk

Adaption to our environment broadly involves selective pressures against an excess of infection in the form of population epidemics and a deficiency of energy in the form of starvation. It follows that we have evolved genetically to resist infections with aggressive immune responses and to resist starvation through energy capture and efficient energy use. These two states are inter-related, since immune responses are energy-consuming responses. It follows that, at the margins, an immune response to an infection can be maladapted by being an exaggerated or deficient response. Autoimmune diseases are considered a consequence of the former, and intriguingly have become more prevalent in the last century [10]. That increase in disease prevalence, even in the last couple of decades and for T1D, is particularly marked in very young age groups. Since the increased risk operates over a short period and within two generations, it is likely that non-genetic factors are responsible. Several theories have been elaborated to explain both the non-genetic effects in T1D and the increasing potency of these effects in recent decades.

### 2.5. Non-Genetic Factors Causing Type 1 Diabetes

Non-genetic factors inducing the disease process are unlikely to operate as intermittent epidemics, given the absence of evidence for epidemics and the importance of age and not time on disease risk. It follows that the non-genetic effect is probably part of a background risk, which operates at critical windows defined by age. Several hypotheses have proposed how genetic and non-genetic factors might operate in the development of T1D. These hypotheses usually invoke the nature of the non-genetic effect, including: the migration hypothesis; the diet hypothesis; the virus hypothesis; and the threshold hypothesis. In general, it will be recognised that all of these hypotheses have some validity, but the fact that they are not considered genetic hypotheses but rather are largely based on the nature of the non-genetic effect is consistent with the known and the unknown, that is, with our extensive knowledge of genes and their potential role, and our limited understanding of non-genetic factors.

### 2.6. Threshold Hypothesis

Given the variable implication of genetic and non-genetic factors, a model based on the hypothesis that genetic and environmental factors operate as intersecting and reciprocal trend lines based on odds ratios results in a method of concurrently evaluating both features and defining the attributable risk for the clinical onset of T1D. One model, the ‘threshold hypothesis’, conceptualises the complex interactions of nurture with nature in T1D [11].

Given that the incidence of T1D has increased rapidly in recent decades, it is notable that the immunophenotype of newly-diagnosed cases has also changed. One study of diabetes carried out between 1985 and 2002 found that the prevalence of Islet Antigen-2 autoantibodies (IA-2A) and Zinc Transporter-8 autoantibodies (ZnT8A) increased significantly in that period, with raised levels of IA-2A, ZnT8A, and IA-2β autoantibodies also seen [12]. IAA and GADA prevalence and levels did not change over the same period. Over a similar period, the genetic risk shifted from high genetic-risk to an increased contribution of moderate-risk genes, in line with a shift in the relative contribution of genetic and non-genetic features, to achieve a threshold for disease induction [12]. Also in line with a threshold effect is the higher the HLA genetic risk the more likely the appearance of multiple autoantibodies and the earlier the age of that appearance. Indeed, in one analysis, multiple autoantibodies appeared in the first 5 years of life, but the single autoantibodies continued to appear for a further 5 years. By implication, the non-genetic effect is likely different in the two processes without being mutually exclusive [13]. Focusing on the group who develop T1D in the first 5 years of life, it does seem likely that one effect is virally driven, given that the odds ratio of developing T1D was substantially greater in those who have viral upper respiratory infections in the first and second 6-month periods of life after birth [14].

### 2.7. Rare Variants 

Genetic predisposition could reside in one or a few potent genes (as in autosomal dominant traits), or many rare variants not easily detected in genetic analyses. In T1D, the evidence is that neither is the case. Rare variants of the gene are variants which expanded in the last 5–10,000 years, and 86% of protein-coding single nucleotide variations arose at that time [15]. These mutations are likely enriched for large effect. In theory, these rare gene variants might be important in autoimmune disease, since there has not been enough time to select them out [16] and given the combined effects of explosive, recent accelerated population growth and weak purifying selection [15]. However, in practice, it is unlikely that rare variants play a major role in autoimmune diseases [17]. It might be argued that many rare variants have a cumulative effect to impact T1D. However, here we are seeking utility in that the variants should ideally define a genetic mutation that has a major effect of the disease pathogenesis so that it can be targeted for disease prevention. The limitations of Genome-Wide Association Studies (GWAS) have been highlighted in a recent report [18]. The authors note the limited biological relevance of GWAS and that many ‘hits’ were in ‘non-coding’ genetic regions. Such ‘non-coding’ regions are potentially in genes coding for elements of regulatory networks at distal enhancers, themselves controlled by epigenetic effects. Were such regulatory networks to be defined, then they might well have clinical utility broadly missing from the current GWAS studies. 

### 2.8. Seasonal Impact of Gene Effect

Many diseases, including T1D, have a seasonal course both in terms of the disease induction and the time of onset of the clinical disease. One study found that more than 4000 protein-coding mRNAs in both white blood cells and adipose tissue have seasonal expression profiles [19]. Importantly, that seasonal variation was inverted between Europe and Oceania. The gene expression periodicity could result from periodicity in the cellular composition of blood by season changes, which differ between Great Britain and The Gambia. It was noted that the immune system had a profound pro-inflammatory transcriptomic profile during the European winter, with increased levels of soluble IL-6 receptor and C-reactive protein; these observations are particularly interesting as autoimmune diseases have peak incidences of clinical onset at that time [19]. However, given the likely window of disease induction in early life, our interest in the seasonal effect of genetic associations should be constrained, as such associations may be relevant to the precipitation of the clinical disease but unlikely to be associated with the disease pathogenesis.

### 2.9. Nature of Genetic Risk

Genes associated with T1D have been well-established in many substantial studies [20,21,22,23]. These genes have four broad functions [24]:

1. Cytokine production/signalling.
2. Decreased T cell signalling/activation.
3. Increased T1 interferon and antiviral response.
4. Antigen presentation and T cell repertoire formation.

Three clusters of genes were identified in a recent GWAS of multiple autoimmune diseases. Of note is that these gene clusters equate broadly with the four functional groups identified above [24].

Cluster 1 genes, such as *ICAM1*, *CD40*, *JAK2*, *TYK2*, and *IL12B*, all with known roles in immune effector cell activation and proliferation, and enriched for genes associated with primary sclerosing cholangitis and inflammatory bowel disease. The expression of these genes was highest in a subset of CD11b dendritic cells.

Cluster 2 genes [21,22] *IL19*, *IL20*, *STAT5A*, and *IL2RA*, which regulate effector T cell activation, differentiation, and proliferation, encoding a number of cytokines and cytokine-response factors. These genes are enriched in multiple sclerosis and expressed in mature natural killer (NK) cells, NK T cells, T cells, and neutrophils.

Cluster 3 genes that encode nucleic acid–binding proteins, such as *ILF3*, *CENPO*, *MED1*, and *NCOA3*. Genes in this cluster are associated with systemic lupus erythematosus [25].

When integrating T1D and GWAS data with protein–protein interactions to construct biological networks, 17 networks were identified [25]. Three networks were enriched for cytokine-regulated genes (as for Cluster 2 above), and eight of these regulated genes (*CD83*, *IFNGR1*, *IL17RD*, *TRAF3IP2*, *IL27RA*, *PLCG2*, *MYO1B*, and *CXCR7*) showed single nucleotide polymorphisms (SNPs) associated with T1D. Other genes implicated in both T1D risk and cytokine action through pro-apoptotic signal transduction includes *HIP14*, *TNFAIP3*, *TYK2* and *CTSH* [22]. As discussed previously, the identification of the networks justifies the generation of GWAS data, though it is never clear from GWAS exactly in which cells the abnormal networks might operate. Defining the site of altered gene networks is possible though Epigenome-Wide Association Studies (EWAS), and these are discussed later.

It is inappropriate to consider T1D as a single disease without variation, since studies indicate potent disease heterogeneity, notably in the rate of β-cell loss, immunogenotypes, responsiveness to immunotherapies, and, in limited studies, in islet pathology [26]. For example, a recent study revealed two distinct types of insulitic lesions distinguishable by the degree of cellular infiltrate and the presence of B cells, termed “hyper-immune CD20Hi” and “pauci-immune CD20Lo”; a multidimensional cluster analysis also showed two equal-sized patient agglomerations characterized by pro-inflammatory (IFN-γ-positive, multi-autoantibody-positive) and partially regulated (interleukin-10-positive, pauci-autoantibody-positive) responses [27]. Multi-autoantibody-positive nondiabetic siblings at high risk of disease progression showed similar clustering. By implication, different immunopathological processes (endotypes) may underlie T1D risk [27]. Set against that interpretation is the substantial evidence that, while there is heterogeneity across the clinical spectrum of T1D based on age at diagnosis, that spectrum does not represent multiple clusters with distinct genetic risk. Instead, the evidence supports similar genetic factors, with the HLA genotypes operating at differential intensity and variable protective effect, while some non-HLA also may impact the genetic risk. In other words, the broad outline of the disease process is similar across a wide age spectrum, but the specific immunogenotypes at any point along that spectrum show variation [26,27,28].

In summary, GWAS have demonstrated many genes associated with T1D, but their aetiological fraction is very variable, with many having little impact on the disease. There is some utility in defining these genes, as they implicate certain networks, and such networks can illustrate the shared genetic nature of autoimmune diseases.

## 3. Specific Genetic Factors and Type 1 Diabetes

### 3.1. Human Leukocyte Antigen (HLA) 

While it is well-established that the main genetic associations with autoimmune diseases involve the HLA class II region [28], though other common variants have been identified [29,30].

Genes encoding HLA molecules are located within the Major Histocompatibility Complex (MHC) on the short arm of chromosome 6. The MHC is divided into class I (HLA-A, -B, and -C), class II (HLA-DR, -DQ, and -DP), and class III (genes for complement components). The class I and class II proteins coded by the relevant genes are transmembrane cell surface glycoproteins, which are critically involved in the presentation of both self and foreign antigens as short peptides to T lymphocytes.

HLA genes are highly polymorphic, with a degree of coding region diversity unequalled elsewhere in the genome. Polymorphisms of certain genes probably originated in selection pressures exerted by environmental factors, including epidemics, climatic change, and availability of food. Non-human somatic studies of the HLA-DQ beta region suggest that this region has been in balanced polymorphism for 10 or more million years, and to maintain the extraordinary diversity of HLA types over this time, selection pressures must have been operating; otherwise most alleles would have been lost through genetic drift. Infectious pathogens are, most likely, the major cause of HLA diversity, and it seems likely that we have adapted our HLA genes to limit the risk of pathogens, most likely ancestral pathogens, with the result that these same genes which adapted to protect us are now disadvantageous. The distribution of sequence variation is clustered in nucleotides, which code for amino acids composing the antigen binding groove. By implication, natural selection must have acted at this binding site to maintain structural diversity for peptide binding. Interestingly, the key HLA genes associated with populations, e.g., Europeans and Chinese, does differ and in the same manner the HLA genes associated with T1D differs in Europeans and Chinese. Such differences may account for the similar autoimmune diabetes risk in adults in the two cohorts, while Europe and China have the highest and lowest incidence of childhood-onset T1D in the world [29,31].

### 3.2. Nature of HLA Risk in Type 1 Diabetes

The evidence is that class II genes are more important than class I genes and that DQ genes are more important than DR genes. The major genes for T1D, HLA-DQB1 and HLA-DRB1 on chromosome 6p21.3, account for approximately 50% of genetic susceptibility [29,30]. T1D incidence, and HLA haplotypes, can vary between ethnic groups [29]. For example, in Caucasians, the DR3 and DR4 haplotypes are prevalent, however, they are rare in Japanese populations [31,32,33,34]. On the other hand, the DR4-DQ (DRB1*0405-DQB1*0401) and DR9 (DRB1*0901-DQB1*0303) susceptible HLA haplotypes are more common in Japanese and are rare in Europeans [34,35].

About 95% of European patients with childhood-onset T1D have either HLA-DR3 or DR4 as compared with about 60% of the normal population. Extended haplotypes identical at the DQB1 loci are associated with very different risks of T1D, implying that MHC genes outside the DQ region play an important role in determining genetic susceptibility [29]. HLA alleles associated with diabetes susceptibility include HLA-DR3, DQB1*0201 and DR4, DQB1*0302. Others are associated with disease protection, e.g., the HLA-DR2, DQB1*0602 haplotype, which occurs in over 20% of some populations, but less than 1% of children who develop T1DM; however, with older age at onset the frequency of the HLA-DR2, DQB1*0602 haplotype is similar to controls, implying its protective role is lost [36].

### 3.3. HLA Protection Associated with Type 1 Diabetes

HLA protection is striking in T1D. Indeed, genetic protection is dominant to susceptibility. The protective effect operates at each stage of the sequence: immune activation; immune persistence; and immune destruction leading to clinical disease. That protection derives from HLA-DQB1*0602, which is rare in children with T1D, but with near normal frequency is comparable to controls with increasing age at diagnosis. Importantly, HLA protection reduces the frequency of multiple autoantibodies, but not autoantibodies in general. It especially reduces IAA, but not GADA. In a recent study, it was found that young relatives with HLA-DRB1*15:01-DQA1*01:02-DQB1*0602 less frequently expressed those autoantibodies associated with higher T1D risk, were less likely to have multiple autoantibodies, and rarely converted from single to multiple autoantibody positivity on follow-up [36]. These relatives also had a reduced frequency of metabolic abnormalities at baseline compared with other relatives, and exhibited no overall metabolic worsening during follow-up. The HLA-DRB1*15:01-DQA1*01:02-DQB1*0602 haplotype seems to reduce the expansion and progression of altered immune response and the co-incident metabolic dysfunction [36]. In other words, the protective influence of the DRB1*15:01-DQA1*01:02-DQB1*06:02 haplotype spans the disease pathogenesis from autoantibody development through all stages of progression. Subjects with this haplotype had a very low 5-year cumulative incidence of T1D. In conclusion, the protective influence of the DRB1*15:01-DQA1*01:02-DQB1*0602 haplotype spans from autoantibody development through all stages of progression, and relatives with this allele only rarely develop childhood-onset T1D. However, importantly, that effect is lost in adulthood, perhaps because in adulthood the dominant autoimmune response is associated predominantly with GADA, and the protection associated with the HLA DRB1*15:01-DQA1*01:02-DQB1*0602 haplotype is not evident in GADA-positive relatives [36].

### 3.4. Structure of HLA-Mediated Risk

The extended haplotype could be important because it codes for the overall structure of the molecule, which forms the binding groove [37,38,39,40,41,42,43,44,45]. Alternatively, it might include other as yet unidentified genes which play a role is disease susceptibility. Certain individual residues confer a particular susceptibility or protection from disease. For example, disease susceptibility correlates with the expression of a DQ molecule bearing an arginine in the 52 position of the alpha chain and lacking an aspartate at the 57 position of the beta chain [37]. A combination of these changes confers a greater risk for disease than either one alone, as has been shown for DR3 and DR4, implying that two or more genes are important in disease susceptibility. On balance, the evidence suggests that certain amino acid substitutions are important, but only within the context of the whole molecule.

These proposals have been recently consolidated in an important study [38]. Using expression quantitative trait locus (eQTL) mapping to test for associations between genetic variation and T cell receptor (TCR) Variable (V) gene usage in a large human cohort, the authors found strong trans associations between variation in the MHC locus and TCR V gene usage. Fine-mapping of the association signals identified specific amino acids from MHC genes that biased V gene usage [38]. Of these, many contacted or were spatially proximal to the TCR or peptide in the TCR–peptide–MHC complex. As a result, MHC variants can directly affect TCR–MHC interaction. This study illustrates how trans-QTL effects can be mediated by protein–protein interactions consistent with intrinsic TCR–MHC specificity [38]. Another important study investigated the role of HLA protection [36,39]. The authors investigated the role of HLA molecules in Goodpasture’s disease, which is an HLA-linked autoimmune disease affecting kidneys and lungs. The disease is characterized by an immunodominant CD4+ T cell self-epitope from the α3 chain of type IV collagen (α3135-145). In Goodpasture’s, HLA-DR15 confers disease risk, while HLA-DR1 is dominantly protective in trans with HLA-DR15, not unlike HLA-DR3 and DR4 predisposing to T1D, while HLA-DQ0602 is dominantly protective. The authors found that HLA-DR15 and HLA-DR1 exhibit distinct peptide repertoires and binding preferences, and present the α3135-145 epitope in different binding registers to promote a cell with a conventional T cell phenotype that secretes pro-inflammatory cytokines [39]. In contrast, T cells with HLA-DR1 in trans are predominantly CD4+Foxp3+ regulatory T cells expressing tolerogenic cytokines. In patients with Goodpasture’s disease, the α3135-145-specific CD4+ T cell repertoire is clonally expanded. By inference, HLA polymorphisms shape the disease-associated CD4+ cells, but can also dominantly protect from disease by enhancing the abundance of self-epitope-specific T regulatory cells. In short, HLA shapes T cell repertoires to account for the yin-yang of causation-protection mediated by HLA polymorphisms.

### 3.5. HLA Loci Heterozygosity and Disease Risk

A striking feature of T1D is the association of gene risk with HLA heterozygosity and the decline in that risk with increasing age at diagnosis and with time. The older the age at diagnosis, the lower the risk of heterozygosity. Moreover, that genetic risk of T1D has changed in recent years so that children with a more moderate HLA-mediated risk, including a reduced frequency of HLA heterozygosity, are at increased risk of developing the disease. Viewed from the vantage of the threshold hypothesis [11] and the discussion above of the HLA shaping the T cell repertoire, it follows that modest genetic susceptibility can lead to the disease. However, the disease is likely to be more severe, as in children, when fewer HLA protective genotypes are in evidence allied to HLA heterozygosity [29,36].

### 3.6. HLA Hyperexpression and Type 1 Diabetes

Human pancreatic beta cells have been implicated in their own demise in T1D, famously encapsulated by Bottazzo in the rhetorical question “homicide or suicide?” [46]. One potentially contributing factor to suicide is the hyperexpression of HLA class I antigens. In a recent investigation of pancreatic HLA class I expression at the protein and RNA level, it was concluded that Islet cell HLA class I hyperexpression is not an artifact [47]. HLA class I hyperexpression was closely associated with an elevated expression of signal transducer and activator of transcription 1 (STAT1), and, together, these occurred uniquely in T1D. Pancreas samples from the Network for Pancreatic Organ donors with Diabetes (nPOD), the Diabetes Virus Detection study (DiViD), and a UK collection were immunostained for HLA class I isoforms and STAT1; hyperexpression of HLA class I was observed in the insulin-containing islets from all three tissue collections, and confirmed at both the RNA and protein levels. The expression of β2-microglobulin (a second component which is required for the generation of functional HLA class I complexes) was also elevated [47]. This hyperexpression did not correlate with detectable upregulation of a transcriptional regulator. The authors concluded that HLA class I hyperexpression contributes to pancreatic beta cells’ selective susceptibility to autoimmune-mediated destruction. A genetic association with the insulin gene, irrespective of the age at diagnosis, also suggests that islet beta cell “suicide” remains an option [39]. However, HLA hyperexpression in cells in conjunction with a genetic change in antigen expression could not only be relevant to islet beta cell death but also to antigen presentation in pancreatic lymph nodes (peripheral tolerance) and the thymocyte expression of potential antigens (central tolerance). The precise battlefield in which these factors come into play remains unclear, and might theoretically be resolved by assessing epigenetic changes.

## 4. Immune Synapse Genes

T1D-risk variants, other than HLA, have been shown to confer risk or protection in several other autoimmune diseases [40,41,42,43,44]. For example, *PTPN22* is involved in T and B cell receptor signaling, and is a risk factor for both Rheumatoid Arthritis and T1D. Another, *CTLA4*, an inhibitory receptor expressed by T cells, is associated with Rheumatoid Arthritis and T1D [20,21]. Over 60 distinct genetic polymorphisms other than HLA alleles have been associated with T1D-risk [22,23]. Genes that associate with a putative antigen (insulin), HLA, and T cell activation implicate the immune synapse. These genes, including HLA, as discussed above, but also *PTPN22* (an allele of the gene for a negative regulator of T cell activation and cytotoxic T lymphocyte antigen 4 (CTLA-4) on chromosome 2q33), are associated with increased levels of soluble CTLA-4, and the frequency of regulatory T cells. Gene polymorphisms within the insulin gene upstream promoter region as well as the *CTLA4*, *PTPN22*, *IRS-1*, *ICOS*, and *SUMO4* genes confer a substantial risk to T1D, with an odds ratio (OR) between 1.8 and 2.5. Of particular note, the *CTLA-4* gene also plays a role in thyroid autoimmunity, and with the HLA region comprises an important molecule in the so-called immunological synapse at the interface of the presentation of an antigen by antigen-presenting cells to immune effector cells. When considered as an immune complex interacting at the immune synapse in the trimolecular complex at the heart of the adaptive immune response, the concurrence of genetic risk mediated by the *HLA* genes, insulin gene, and *CTLA4* and *PTPN22* genes strongly implicate disease risk operating at this immune synapse to induce an altered immune response [45].

### 4.1. Insulin Gene Plus Insulin as Antigen

The Insulin-Dependent Diabetes Mellitus 2 (*IDDM2*) locus, also known as the insulin gene (*INS*) region, contributes about 10% toward T1D susceptibility [48,49,50,51,52,53] and is found on chromosome 11p15.5 [51]. This locus has variable number of tandem repeats (VNTR), a type of mini-satellite. There are three classes of VNTRs that are divided by the number of repeats. Class I VNTRs are 570 base pairs (bp) long, class II 1200 bp, and class III 2200 bp [49]. Class I is the shortest of the three and is associated with a high risk of developing T1D, whereas class III provides a more dominant protective effect [46].

Insulin genes can contribute to disease penetrance. In a twin study investigating two susceptibility genes in T1D, the *INS* and HLA-DQB1, we found that a particular *INS* genotype (Hph I) was identified in 88% of the concordant twins but only 60% of the discordant twins [53]. This suggests that the possession of the high-risk haplotype increases the likelihood of MZ twins becoming concordant for T1D.

The increased expression of insulin (mRNA) in the thymus of people with “long” or protective repeats—which suggests a more efficient deletion of insulin-specific T cells during the induction of central tolerance—provides an attractive potential mechanism for the role of the insulin gene in T1D. This hypothesis also provides an alternative site (the thymus mTEC cells) for antigen (insulin) and immune synapse engagement other than the peripheral blood, pancreatic lymph nodes, or pancreatic islets. However, the difficult task of examining such mTECs from the thymus in children at risk of T1D is yet to be performed, and may pose an insuperable challenge. In a recent study of patients with non-insulin requiring autoimmune diabetes (also called latent autoimmune diabetes of adults (LADA)), it was found that the insulin gene risk alleles were as prevalent in LADA as in childhood-onset T1D, consistent with insulin-related pathways playing an important role in T1D irrespective of the age at onset or mode of onset [54].

### 4.2. Interferon (IFN) Genes and Virus Infections

Class 1 hyperexpression is a feature of islets in patients with T1D, as previously noted. Class 1 hyperexpression (HLA plus beta 2 microglobulin upregulated) plus STAT1 expression in islets of patients with T1D are consistent with an interferon effect (though importantly it affects islets both with insulitis and those without insulitis) [47,55].

The type I IFN response, which results in the expression of diverse IFN-stimulated genes, is known to contribute to the control of viral infections. Type 1 IFN signalling is also involved in the establishment of persistent viral infections and their associated immune suppression. Gene networks and the loci underlying their regulation have been identified in an interferon regulatory factor 7 (IRF7)-driven inflammatory network enriched for viral response genes [55,56,57,58].

These variants are predicted to alter the expression and structure of IFIH1 (MDA5), a cytoplasmic helicase that mediates the induction of interferon response to viral RNA. T1D risk in a cohort of DAA revealed that diabetes risk was associated with the IFIH1 rs2,111,485 SNP, but did not associate with the development of islet autoantibodies or autoantibodies against thyroid peroxidase and tissue transglutaminase [57]. Children with the IFIH1 rs2,111,485 GG genotype had a faster progression to diabetes (31% within 5 years) than children with the T1D protective GA or AA genotypes (11% within 5 years). The findings indicated that IFIH1 genotypes impacted progression from autoimmunity to diabetes development, which is consistent with the notion that protective genotypes downregulate responses to environmental insults even after the initiation of autoimmunity [36]. Importantly, these observations imply a viral effect on the development of T1D irrespective of the age at diagnosis, so the evidence that early events causing childhood-onset T1D are likely to include viral effects can most likely be extended to those in whom the disease induction is later. However, the question remains: how early do these genetically mediated changes develop? Two groups found that genes and pathways related to innate immunity functions, such as the type 1 IFN response, are active early in life and before DAA are detected, with IFN response factors identified as central mediators of the IFN-related transcriptional changes. Indeed, perinatal factors (Caesarean section) are related to disease risk and IFN-associated genetic risk; increased T1D risk was even seen in children delivered by caesarean section with IFIH1 genotypes (12-year risk, 9.1% vs. < 3% for all other combinations) [58].

### 4.3. Gut and Genetic Susceptibility to Type 1 Diabetes

The gut microbiome has been implicated in human disease, including autoimmune diseases [59,60,61,62,63]. Of particular note in the context of disease is the loss of gut microbiome diversity and the preponderance of certain pro-inflammatory genera. In one study of children at genetic risk of T1D, it was found that at-risk children with diabetes autoantibodies had an increased abundance of Bacteroides and a reduced abundance of Prevotella in the gut microbiome compared with those at lower T1D risk due to having only one diabetes-associated autoantibody [57]. The abundance of Bacteroides was of particular note given the studies of the bacterial lipopolysaccharide from Bacteroides, which found that it was structurally different from that of *Escherichia coli*, less pro-inflammatory, and less likely to reduce disease progression in animal models of diabetes [61,62], while those with no autoantibodies or healthy controls had a distinct gut microbiome consistent with the gut flora being linked with disease risk. Furthermore, those subjects that progressed to T1D showed a marked drop in gut flora diversity in the time window between developing disease-associated autoantibodies and developing clinical T1D [59,60]. If the gut flora is relevant, what can be done to modify that flora? One option would be to alter the diet. There has been much interest in dietary modifications to reduce the risk of T1D, and a detailed analysis is beyond the scope of this review. However, a recent study did seek an association between early probiotic intake and T1D risk in a large cohort of children aged 4 to 10 years of age carrying HLA disease risk genotypes [63]. It was found that probiotic supplements in the first 27 days of life were associated with a reduced risk of progression to diabetes-associated autoantibodies, but only in those with high genetic risk, i.e., HLA 3/4 heterozygosity [63].

## 5. Genetic Effect on the Variable Clinical Presentation

We have described the tendency for the disease to progress more rapidly in some patients as compared with others in those with high HLA-mediated genetic risk and multiple autoantibody positivity. There is now substantial evidence that non-HLA genes also play a role in such progression.

### 5.1. Non-HLA Genes Impacting Disease Progression

In one study, children born to a parent with T1D were prospectively followed from birth for up to 22 years and examined for the frequency of non-HLA genes [64]. In this cohort with multiple autoantibodies, rapid and slow progressors were similar with respect to the HLA-DR/HLA-DQ genotypes, and IA-2A development was considerably delayed in the slow progressors, while the groups were distinguished by the presence or absence of non-HLA genes, most notably *IL2*, *CD25*, *INS VNTR*, *IL18RAP*, *IL10*, *IFIH1*, and *PTPN22*. In support of that observation, in a Finnish study of 1650 children that were followed, 23 developed multiple autoantibodies and progressed to diabetes within 3 years (rapid progressors), while 24 children developed multiple autoantibodies and remained non-diabetic for more than 10 years from seroconversion (slow progressors); it was found that the *PTPN22* 1858T allele, but not the *INS-23* HphI AA genotype, was associated with progression [65].

In a large European study, nearly 15,000 patients were genotyped at confirmed T1D-associated non-HLA regions to seek interaction of association with age at diagnosis, sex, and HLA class II genotypes [66]. The alleles that confer susceptibility to T1D at *IL-2*, *IL2/4q27* (rs2,069,763) and renalase, flavin adenine dinucleotide (FAD)-dependent amine oxidase (*RNLS*)/10q23.31 (rs10,509,540), were associated with a lower age at diagnosis (more rapid progressors) with individuals carrying the susceptible homozygous genotype being, on average, 7 months younger at diagnosis than those carrying the protective homozygous genotypes [66]. These results are consistent with a non-HLA effect causing T1D and impacting its age at diagnosis [66,67].

### 5.2. Low Risk HLA Genes and Single Autoantibodies

Single autoantibodies, usually GADA, can appear at any age and their frequency, in contrast to multiple autoantibodies, increases in at risk children up to, and even after, 10 years of age, at which state they continue to carry an increased risk of progression to clinical diabetes [68]. The genetic loci most strongly related to T1D in children are those also found in adults with T1D, while their genetic risk includes similar loci with the same directional effect but with a lower Hazard Ratio [69]. The HLA-DRB1*03/HLA-DRB1*04 (DR3/4) genotype effect in such adult-onset T1D is still high (odds ratio 26.22), while DR3/4 and DR4, but not DR3, are associated with a later age at diagnosis. Of particular note, the HLA protective alleles, almost absent in childhood-onset T1D, have a similar frequency to the control population in adult-onset T1D [69]. Taken in the round, these results are consistent with the twin data, which indicates that the heritability of T1D declines progressively with increasing age at diagnosis. By implication, the non-genetic aetiological fraction should increase in those presenting with T1D as adults, while the genetic effect should decrease, and that is what is found with the known genetic loci.

### 5.3. Immunogenotype and Adult-Onset Diabetes

Multiple autoantibodies and strong HLA risk are associated with a severe loss of insulin secretory capacity and rapid progression to diabetes. As the HLA risk declines, so do the number of autoantibodies, especially IA2A, and the level of insulin secretion at diagnosis is higher. A striking feature of the architecture of autoimmune diabetes is that there is a quantitative effect, consistent with the threshold hypothesis, so that a sizeable proportion of cases present as adults with non-insulin requiring diabetes and appear clinically to have Type 2 diabetes. These patients are defined by the presence of DAA, usually GADA, and therefore their autoimmune diabetes is latent until DAA are checked. They have been called, amongst many acronyms, latent autoimmune diabetes of adult-onset (LADA); the immunogenotype of LADA is similar to T1D [5,31,54,70]. A Chinese study of LADA was striking because T1D in childhood is remarkably rare in China (the lowest frequency in the world) [31]. A nationwide Chinese multicentre, clinic-based cross-sectional study identified 4880 ketosis-free diabetes patients (<1 year post-diagnosis, without insulin therapy for more than 6 months and aged more than 30.0 years), of whom 5.9% were GADA-positive and designated LADA, and these LADA patients had HLA diabetes-susceptible haplotypes, while HLA diabetes-protective haplotypes were less frequent than the Type 2 diabetes patients who did not have GADA [31].

A Scandinavian study of patients with LADA (n = 911) or Type 2 diabetes (n = 406), all diagnosed after the age of 35 years, identified variants in the *ZMIZ1* (rs12,571,751) and *TCF7L2* (rs7,903,146) loci strongly associated with LADA [70,71]. Variants associated with Type 2 diabetes were strongest in patients with low GADA, raising the question of whether a proportion of cases had Type 2 diabetes with false positive GADA (as the assay for GADA had 95% specificity). Since LADA and adult-onset T1D could share genetic risk variants with Type 2 diabetes, this issue is now being explored in more detail. A preliminary study by ourselves failed to confirm the *TCF7L2* (rs7,903,146) association, while it did confirm that LADA is predominantly T1D in terms of genetic risk, albeit at lower risk allele frequency than in childhood-onset T1D [54]. Specifically, HLA apart, known T1D genetic loci were strongly associated with LADA, including *PTPN22*, *SH2B3*, and *INS*. By constraining the data set on those with multiple (two or more) DAA, the similarity between LADA and T1D became more apparent [54].

**Figure 3:**
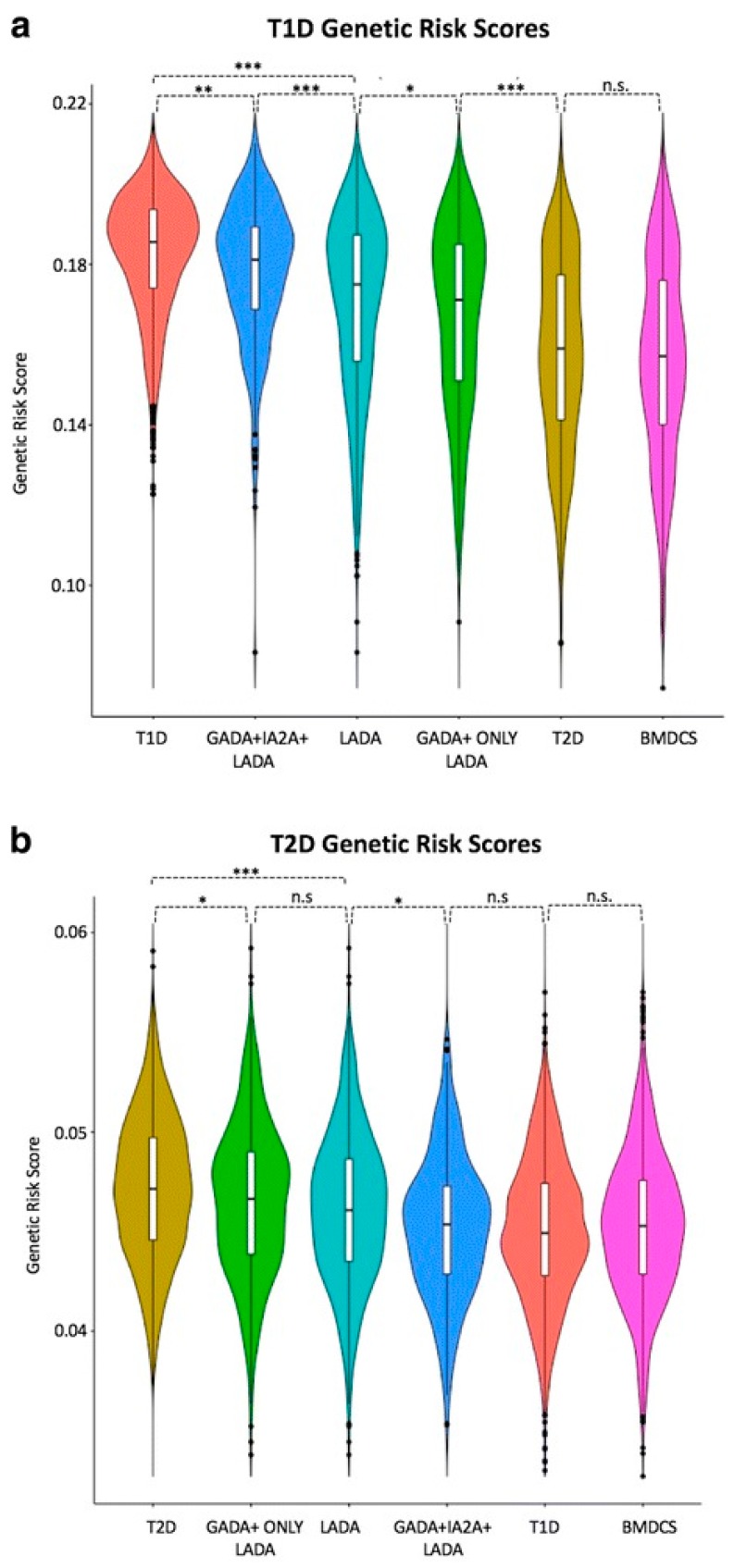
Genetic risk score (GRS) between T1D, Type 2 diabetes (T2D), latent autoimmune diabetes in adulthood (LADA), and LADA-restricted cases and controls. The GRS distributions were compared between subjects with T1D (n = 1990), T2D (n = 1960), LADA (n = 978), LADA restricted (n = 309), LADA GADA-only (n = 669), and Bone Mineral Density in Childhood Study (BMDCS) controls (n = 1057). The Violin plots show the distribution of GRS for the five groups: (**a**) using the T1D single nucleotide polymorphisms (SNPs) for the five groups and (**b**) using the T2D SNPs. A multiple comparison test (Wilcoxon rank sum test) was performed to calculate pair-wise differences, and key differences are highlighted. (*** *p* < 0.00001, ** *p* < 0.0001, * *p* < 0.05) [54].

Only one Type 2 Diabetes (T2D) locus, HNF1A, was associated with LADA, but that association was weak needs to be confirmed [54]. Of particular note, the T2D genetic risk score (calculated by adding the log of the odds ratio for each of 69 SNPs for a given individual) was higher in patients with LADA in general than those with LADA constrained by having two DAA (Figure 3) [54]. By implication, there may be some T2D gene risk alleles in LADA.

Two points arise from these observations. If we reflect on the nature of autoimmune T1D, it is clear that with increasing age at diagnosis there is decreasing genetic risk and decreasing disease severity. It is logical to assume that with a modest loss of insulin secretory capacity, additional factors that predispose to reduced insulin secretion and insulin sensitivity would predispose to clinical diabetes. Those factors could be genetic, including genes associated with the loss of insulin secretion and insulin sensitivity, or non-genetic, for example, aging and increasing diet-related obesity. Since even childhood-onset T1D is strongly associated with the national Gross Domestic Product [69], it is highly probable that non-genetic factors play a major role in the development of adult-onset autoimmune diabetes.

### 5.4. Immune-Mediated Destruction and Diabetes

From the data we have considered, we can surmise that the destructive process in T1D is likely to be immune-mediated and associated with an adaptive immune effect based on the association with HLA loci and DAA. However, the presence of DAA in a substantial fraction (~10%) of adults with non-insulin requiring diabetes has raised issues regarding the specificity of the antibody assays and the validity of the concept of LADA. Antibody assay results could be true of false whether they are positive or negative. 

If we consider an autoantibody result (say GADA) that is positive in an assay that has 95% specificity in a population in which 5% of subjects have GADA, then 50% of the positive cases could be false positives given that true specificity is 1-test specificity. On this basis, it has been argued that the concept of LADA is grossly exaggerated. However, the GADA assays are based on only 1% of a general population having a positive test. The discrepancy is because 5% of the general population do not have GADA, and instead a frequency of 5% derives from patient selection. There will be a proportion of diabetes cases, likely less than 2%, in whom there is false positive GADA, but the frequency of LADA, by implication, is not grossly inflated using the current definition.

False negatives will also be a feature in a cohort analysis. That is, patients without GADA could be negative because they lost the antibody (putatively 60% of GADA positive cases revert to negative), or because the assay falls short of 100% specificity. If either of these were true, then the frequency of LADA cases would be higher than reported. We have currently no way of knowing the extent of this effect, though antibody assays tend to have assay sensitivity well short of 100%.

We conclude that LADA is essentially a form of T1D (we have described it as Type 1.2), with a less aggressive metabolic impact, often single autoantibodies (usually GADA), a reduced genetic risk (both HLA and non-HLA), and normal genetic protection HLA alleles (infrequent in childhood-onset T1D). LADA is prevalent, accounting for 5–15% of patients with adult-onset diabetes.

## 6. Unknown: Features that Could Be Genetically Determined

Evidence that pancreas weight is less in both T1D and individuals at risk of T1D raises the question as to whether these changes might develop early in life, and if so, whether they are genetically or non-genetically determined, e.g. through poor nutrition [72,73]. It is known that the formation of the human beta cell population does get set in early life. In one study, human pancreatic sections derived from normal cadavers aged 24 weeks premature to 72 years of age were examined by immunofluorescence [74]. Most β cell neogenesis was observed preterm, with a burst of β cell proliferation peaking within the first two years of life. Thereafter, there was little indication of β cell growth. The authors concluded that the human β cell population is established before five years of age [75]. This early period, then, is the phase in which the T1D process starts to operate, and the islet cell mass, which could be genetically determined, could itself determine, in part, the time to clinical diabetes.

### 6.1. Stratification of Genetic Risk and Gene Risk Scores (GRS)

The problem with discriminating T1D, whether insulin-dependent or non-insulin requiring (LADA), from Type 2 diabetes and from Maturity Onset Diabetes of the Young (MODY), can be helped by assessing the relative genetic contribution to each. These gene risk scores (GRS) can be made by testing a series of genes and summing the log score of the odds ratio for each gene for a given disease. The higher the score the greater the genetic risk, and as the sum of that risk is a continuum, it offers the possibility to distinguish cohorts with a specific genotype but overlapping clinical phenotypes. A recent study found that GRS could distinguish clinically defined T1D and Type 2 diabetes from the Wellcome Trust Case Control Consortium, and that the T1D GRS correctly classified young adults (diagnosed at 20–40 years of age, an age with maximum diagnostic difficulty in clinical practice) who progressed to severe insulin deficiency a few years from diagnosis [74]. This discrimination was performed using a 95th centile for T1D to exclude most T2D cases, and a 5th centile for T1D to include most T1D cases, but the sensitivity as a predictor was relatively low consistent with the need for several biomarkers to categorise diabetes types. Remarkably, the specificity for this cohort in terms of the prediction of subsequent insulin requirement could be achieved by using only nine SNPs. It remains to be determined how these polymorphisms perform when studying a cohort, in which the genetic load is less strong, and the risk of progression to insulin dependency within 3 years much less than 100%.

The potential of GRS to classify diabetes is in its infancy, but there is much hope that it can promote and assist our understanding of difficult cases, including LADA, Type 2 diabetes in children, maturity onset diabetes of the young, and perhaps other states of diabetes, including pancreatitis. For example, a GRS at 50th T1D centile cut-off in 48 patients with no known genetic cause identified those most likely to have a novel monogenic aetiology identifying probable early-onset T1D (GRS >50th T1D centile) compared with those with established monogenic diabetes [76]. Meanwhile, a separate study investigated established T1D and T2D genetic loci in a large cohort of patients with LADA [54]. In identifying a greater genetic similarity between LADA and T1D than between LADA and T2D, this latter study further highlights the potential use for genetic risk scores in defining subtypes of diabetes (Figure 3) [54].

#### 6.1.1. Potential Genetic and Non-Genetic Interactions

The discordance status of T1D within monozygotic twin pairs points to the importance of environmental factors in that disease. Given the frequency of thyroid autoimmunity in T1D, the question arises as to whether the environmental factor causing T1D is the same as that causing thyroid autoimmunity. In a study of MZ and dizygotic twins discordant for T1D tested for thyroid peroxidase autoantibodies (TPOA), TPOA positivity was higher in females than males, but did not segregate with T1D, with a heritability estimate of 61%. Environmental factors associated with T1D were not the same as those involved with thyroid autoimmunity. Importantly, the implication is that it is just as relevant to study TPOA in the first-degree relatives of patients with T1D as in the patients themselves.

T1D genetic susceptibility loci are shared with other autoimmune or immune-mediated diseases that co-segregate in families with T1D. In a recent study, fifty SNPs were genotyped in 6556 multi-ethnic cases collected by the T1D Genetics Consortium and tested for DAA and autoantibodies against TPOA in autoimmune thyroid disease, gastric parietal cells in autoimmune gastritis, transglutaminase in celiac disease, and 21-hydroxylase (21-OHA) in autoimmune hypoadrenalism. In addition to the MHC region, SNPs in five susceptibility loci (*IFIH1*, *PTPN22*, *SH2B3*, *BACH2*, and *CTLA4*) were significantly associated with more than one autoantibody [77].

#### 6.1.2. Epigenetic Effects and T1D Risk

Epi- is the Ancient Greek term for ‘above’ as in epiphenomenon. Thus, epigenetics is the study of the marks ‘above’ the genes, that is the chemical tags (including methylation and acetylation) on DNA and RNA. It can be envisaged that these marks illustrate a process whereby non-genetic effects can alter gene transcription and translation. In a simplistic understanding of epigenetics, it might be said that epigenetics explains the difference between your ear and your nose (which have identical DNA) as it does the differences between an MZ twin pair (which also have identical DNA).

Several features suggest that T1D could be subject to epigenetic effects (5, 20, 78): (1) MZ twins have a high discordance rate, especially when they are aged more than 15 years at diagnosis; (2) the risk of T1D has increased in recent years more rapidly than could be accounted for by genetic changes alone; (3) the risk for the offspring of a father with T1D is more than that risk for a mother with T1D (6% vs. 1% respectively); and (4) HLA haplotype sharing does not account for diabetes risk, instead it is age at diabetes onset which determines that risk. By implication, non-HLA genes or epigenetic/environmental factors that accelerate the progression of T1D in the proband strongly affect risk in siblings [5,9].

Direct studies of epigenetic changes have implicated changes associated with insulin secretion and diabetes risk [78]. A genome-wide DNA methylation quantitative trait locus (mQTL) analysis in human pancreatic islets was performed using 574,553 SNPs with genome-wide DNA methylation data of 468,787 CpG sites targeting 99% of genes in islets from 89 donors. The authors found that 383 CpG sites (0.08% of tested CpGs) showed significant associations after correction for multiple testing, including known diabetes loci, e.g., *ADCY5*, *KCNJ11*, *HLA-DQA1*, *INS*, *PDX1*, and *GRB10*. The CpGs of significant cis-mQTLs were over-represented in the gene body and outside of CpG islands. Causal inference tests identified SNP-CpG pairs with DNA methylation in human islets as potential mediators of the genetic association with gene expression or insulin secretion. Functional analyses further demonstrated that identified candidate genes (*GPX7*, *GSTT1*, and *SNX19*) directly affect key biological processes, such as proliferation and apoptosis, in pancreatic β cells. The study showed that genome-wide genetic and epigenetic variation can interact to influence gene expression, islet function, and potential diabetes risk in humans.

The problem with these studies of epigenetics is their limited power because of the enormous numbers of CpG sites identified. Using a dense genotyping of autoimmune disease, it was found that T1D is more similar genetically to other autoantibody-positive diseases, most particularly to juvenile idiopathic arthritis, while least significantly to ulcerative colitis. T1D SNPs localised to enhancer sequences in thymus, T, and B cells, and CD34+ stem cells, and this illustrates the power of epigenetic analysis to identify those cells which are actively using the genes associated with a given tissue, given that all cells contain every gene: a state that genetics alone cannot resolve [79]. Recent epigenetic studies used arrays such as 450K by Illumina, in which the distal enhancer regions were excluded. Bisulphite sequencing (very expensive) and the new arrays (e.g., EPIC) can now define enhancer DNA methylation. However, the importance of this shortfall in not defining enhancer region DNA methylation in some of the earlier studies is illustrated by evidence that the causal variants of T1D are found in open-reading frames in the gene enhancer regions of CD4, CD8, and CD14 cells [20]. 

**Figure 4:**
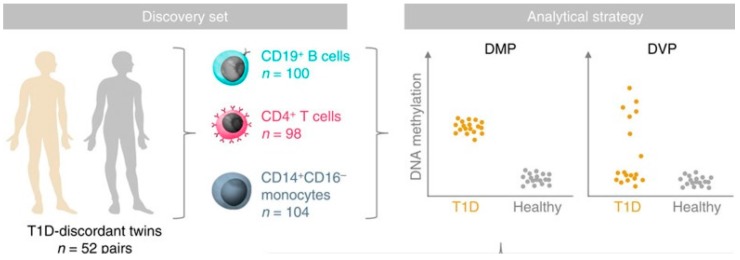
We used (MZ twins discordant for T1D in three different cell types, to define either differentially methylated CpG positions (DMPs), or differentially variable CpG positions (DVPs), as illustrated [80].

An epigenome-wide association study in 52 MZ twin pairs discordant for T1D in three immune effector cell types: CD4+ T cells, CD19+ B cells, and CD14+CD16- monocytes used the Illumina 450K array noted above (Figure 4) [80]. By using disease-discordant MZ twins, our strategy reduced major confounding effects, such as inter-individual genetic variability and in utero effects. There was a remarkable concordance between twins of each pair consistent with a strong shared genetic/non-genetic effect on CpG methylation. There was also substantial enrichment of differentially variable CpG positions (DVPs) in T1D twins compared to their healthy co-twins across all cell types. Compared to the healthy, unrelated individuals, patients with T1D showed cell type-specific enrichment with changes which were temporally stable and enriched at gene regulatory elements. Cell type-specific gene regulatory circuits highlighted pathways involved in immune cell metabolism and the cell cycle, including mTOR signaling. Evidence from the cord blood of newborns who progressed to overt T1D suggests that the DVPs likely emerged after birth [80]. These results implicate epigenetic changes that could contribute to disease pathogenesis in T1D, but further studies of prediabetes subjects using arrays including distal enhancer regions should be more informative, and it remains possible that some of the epigenetic changes found are secondary to the diabetes process and not disease risk.

The MZ twin study was part of a set of studies by the BLUEPRINT consortium, and another study from the same consortium extended those observations [80,81]. The authors studied both DVPs and hypervariable expression (using RNA-Seq) in monocytes, neutrophils, and naïve T cells in healthy individuals. There was a strong relationship between DNA methylation and gene expression. The DVPs were increased substantially in neutrophils compared with the other cells, and these DVPs were enriched at active enhancer and dynamic chromatin regions. By implication, DVPs represent an increased plasticity of normal cells, potentially to deal with cell stress, and that hypervariability is also a feature of established T1D [80,81]. These results could be of value in our understanding of activated cells.

Of the many areas of potential interest and value, the study of microRNAs (miRNAs) has attracted attention, as they can be detected in serum. A recent study was reported of 93 such miRNAs in the serum of 150 children who were at high disease risk, as they had multiple DAA, and a similar number of low disease risk children without DAA [82]. It was found that 15 of these 93 miRNAs were higher or lower in the disease risk children, but that the disease’s sensitivity and specificity as a predictor was limited. Nevertheless, similar approaches, currently in the earliest phase of development, may well prove informative in the future.

## 7. Conclusions

T1D, like other autoimmune diseases, is a composite of genetic and non-genetic effects, leading to the destruction of insulin-secreting cells. However, the variability in presentation of the disease and the presence of distinct immunogenotypes points to heterogeneity reflected in a heterogeneity of the underlying genetic susceptibilities. This heterogeneity is particularly noted in the age at onset of T1D, in which the HLA load is greater with an earlier disease onset.

If genetic and epigenetic analyses are to have clinical utility, it will likely be in disease prediction, prediction of disease outcome, and prediction of best therapeutic approaches; and in this, autoimmune T1D is no different from Type 2 diabetes [83]. Much of what we have discussed relates to disease prediction, given that the combination of genetic risk plus diabetes-associated autoantibodies (due to gene-environment interaction) is a powerful predictor of clinical disease. Less certain, and not discussed, is the potential for genes to predict the macrovascular and microvascular consequences of the disease. However, the genetic risk factors are likely to be shared with T2D, just as are the complications of diabetes. Since T1D and T2D are genetically distinct, the genetic susceptibility to these complications is unlikely to reside within genes associated with the disease risk. The same may not be true for the management of the disease, as the genetic evidence points towards disease heterogeneity, and by implication different approaches may be required to prevent or limit the progression of T1D based on the variable genetic predisposition described here.
